# Association between gut microbiota and spinal stenosis: a two-sample mendelian randomization study

**DOI:** 10.3389/fimmu.2024.1360132

**Published:** 2024-04-19

**Authors:** Jian Li, Jinpeng Wei, Jiani Wang, Tao Xu, Baofeng Wu, Shuhan Yang, Shaoze Jing, Hua Wu, Haihu Hao

**Affiliations:** ^1^ Department of Orthopedics, Third Hospital of Shanxi Medical University, Shanxi Bethune Hospital, Shanxi Academy of Medical Sciences, Tongji Shanxi Hospital, Taiyuan, China; ^2^ Department of Pediatric Medicine, Shanxi Medical University, Taiyuan, China; ^3^ Department of Orthopedics, Tongji Hospital, Tongji Medical College, Huazhong University of Science and Technology, Wuhan, China; ^4^ First Clinical Medical College, Shanxi Medical University, Taiyuan, China

**Keywords:** two-sample mendelian randomization, gut microbiota, spinal stenosis, causal inference, single nucleotide polymorphism

## Abstract

**Introduction:**

Considerable evidence has unveiled a potential correlation between gut microbiota and spinal degenerative diseases. However, only limited studies have reported the direct association between gut microbiota and spinal stenosis. Hence, in this study, we aimed to clarify this relationship using a two-sample mendelian randomization (MR) approach.

**Materials and Methods:**

Data for two-sample MR studies was collected and summarized from genome-wide association studies (GWAS) of gut microbiota (MiBioGen, n = 13, 266) and spinal stenosis (FinnGen Biobank, 9, 169 cases and 164, 682 controls). The inverse variance-weighted meta-analysis (IVW), complemented with weighted median, MR-Egger, weighted mode, and simple mode, was used to elucidate the causality between gut microbiota and spinal stenosis. In addition, we employed mendelian randomization pleiotropy residual sum and outlier (MR-PRESSO) and the MR-Egger intercept test to assess horizontal multiplicity. Cochran’s Q test to evaluate heterogeneity, and “leave-one-out” sensitivity analysis to determine the reliability of causality. Finally, an inverse MR analysis was performed to assess the reverse causality.

**Results:**

The IVW results indicated that two gut microbial taxa, the genus *Eubacterium fissicatena* group and the genus *Oxalobacter*, have a potential causal relationship with spinal stenosis. Moreover, eight potential associations between genetic liability of the gut microbiota and spinal stenosis were implied. No significant heterogeneity of instrumental variables or horizontal pleiotropy were detected. In addition, “leave-one-out” sensitivity analysis confirmed the reliability of causality. Finally, the reverse MR analysis revealed that no proof to substantiate the discernible causative relationship between spinal stenosis and gut microbiota.

**Conclusion:**

This analysis demonstrated a possible causal relationship between certain particular gut microbiota and the occurrence of spinal stenosis. Further studies focused on the mechanism of gut microbiota-mediated spinal stenosis can lay the groundwork for targeted prevention, monitoring, and treatment of spinal stenosis.

## Introduction

Spinal stenosis, a multifactorial disease, is characterized by the narrowing of the spinal canal, which may occur from exogenous factors like trauma, infections, and tumors, as well as endogenous factors like natural degeneration (1). These factors can change the anatomical structure surrounding the spinal canal (posterior longitudinal ligament, ligamentum flavum, and facet joints) and degenerate the intervertebral discs, leading to symptoms such as ow back pain, leg pain, and intermittent claudication (2). As a result of the aging population, an estimated 100 million individuals worldwide are reported to suffer from spinal stenosis, seriously affecting the quality of life for patients and their families and contributing significantly to the global medical and financial burden (3, 4). Though the function of the existed treatment strategies, to develop high-efficiency and fast prevention, treatment and monitoring strategies is very instant.

As the evolvement of the human health research, studies on the gut microbiota have steadily moved to the center of attention (5). Gut microbiota, including prokaryotes (bacteria and archaea), eukaryotes (fungi, intestinal protozoa, and parasitic helminths), viruses, and phages, forms the largest, most important and diverse micro-ecosystem among the digestive ecosystems (6). Due to the diversity and specificity of its structure, the gut microbiota is essential for maintaining a dynamic balance between the intestinal environment and human systems (7, 8). In addition, gut microbiota and its metabolites are involved in the disease process of various organs inside and outside the intestine (e.g., brain, liver, and colorectum). Especially in some metabolic diseases, changes in the abundance of gut microbiota have become a potential risk factor for inducing inflammatory cytokines (9–11). In recent years, there has been growing evidence with regard to the relationship between gut microbiota and spinal degenerative diseases. Specifically, dysbiosis may affect the health of spinal structures through three mechanisms: (1) nutrition, including calcium, amino acids, and vitamin K; (2) immune regulation, such as estrogen, short-chain fatty acids, and systemic inflammation; and (3) neurotransmitters, including serotonin and leptin, which affect bone metabolism (12). Furthermore, the publication by A et al. serves as an overview of earlier research findings and creatively brings up the concept of the intestinal-spinal axis, thus further illustrating the potential relationship between gut microbiota and spinal degenerative diseases (13).Based on the known interactions between the gut microbiota and diseases, several studies have implicated that the occurrence and development of such diseases can be prevented by precisely and simply regulating the biological abundance of gut microbiota (14), providing a new scheme for spinal stenosis prevention, treatment and monitoring.

Thus, comprehending the connection between the gut microbiota and spinal stenosis is essential. Nevertheless, recent reports have only demonstrated some indirect relationships. It is commonly recognized that spinal stenosis is associated with reduced space available for the neural and vascular elements of the lumbar spine (15). As previously mentioned, dysbiosis of the intestinal flora may affect spinal structures (bone, cartilage, intervertebral discs, ligaments, and muscles) through a range of possible mechanisms, such as immunological and nutritional, leading to the development of vertebral osteophytes, lesser articular eminence hyperplasia and hypertrophy, disc herniation, hypertrophy of the ligamentum flavum, and more. And importantly, when the alterations happen in paravertebral structures, causing the volume of the spinal canal to narrow, it can lead to spinal stenosis (16). Also, the correlation between gut microbiota and disorders associated with chronic inflammation and the validation observed in ligamentum flavum thickening raise the prospect of a relationship between gut microbiota and spinal stenosis (17). However, no direct cause-and-effect association between gut microbiota and spinal stenosis has been reported. Comprehending and clarifying the causal relationship between the two mentioned above can offer novel approaches towards the targeted prevention and management of spinal stenosis.

Employing genetic variants to generate instrumental variables (IVs) for exposure and incorporating collected data from genome-wide association studies (GWAS), mendelian randomization (MR) studies offer a whole new approach to evaluate causal relationships between exposures and outcomes (18). At present, MR studies have been widely used to explore the causal relationship between gut microbiota and diseases, including metabolic diseases, autoimmune diseases, and various types of bone diseases (19). Considering the fact that genotypes are randomly assigned from parents to children, it is unlikely that environmental influences will affect the relationship between genetic variation and outcome (20). Therefore, MR research offers an economical and effective approach to investigating the causal relationship between exposure and the outcome. In this paper, a two-sample MR analysis was performed to assess the causal relationship between gut microbiota and spinal stenosis using the GWAS summary statistics from the MiBioGen and FinnGen consortia.

## Materials and methods

**Figure 1:**
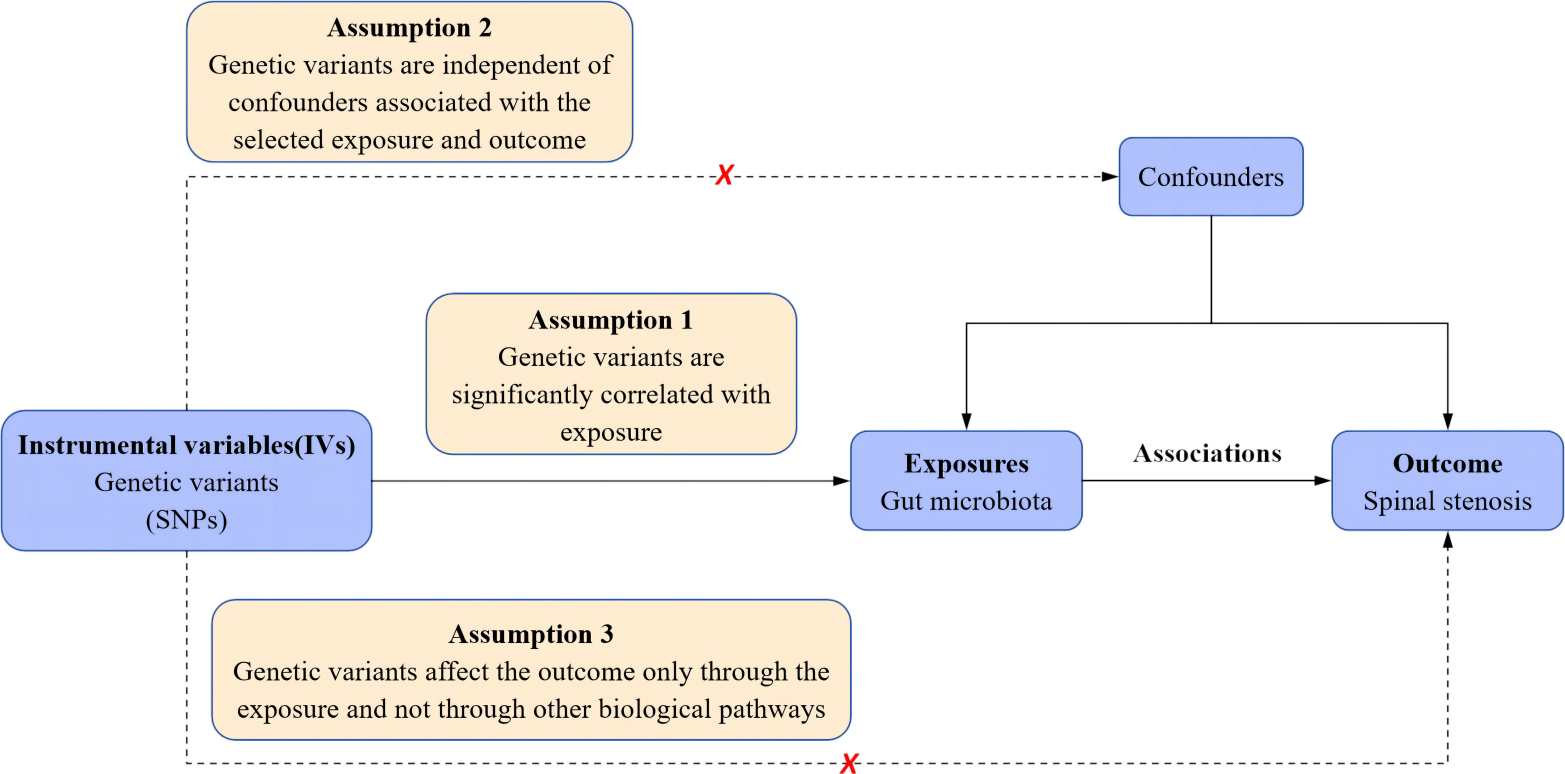
Schematic representation of the a two-sample mendelian randomization analysis. SNPs, single nucleotide polymorphisms.

Based on two-sample MR analyses, we obtained pooled data from published GWAS to evaluate the causal relationship between gut microbiota and spinal stenosis. This study first determined whether gut microbiota contributes to the prevention or promotion of spinal stenosis by selecting gut microbiota as the exposure and spinal stenosis as the outcome. In addition, three crucial presumptions must be met to allow for MR research to proceed: 1. Genetic variation should be significantly linked to exposure; 2. Genetic variation should be independent of confounders associated with selected exposures and outcomes; and 3. Genetic variation can affect outcomes only through exposure and not through other biological pathways (i.e., no horizontal multiplicity of effects) (21). Specific details are shown in **Figure 1**.

### Exposure data sources

The MiBioGen consortium, conducting the largest genome-wide meta-analysis on gut microbiota composition, provided genetic variations of gut microbiota in this study. The meta-analysis examined the relationship between human autosomal genetic variation and the gut microbiome by analyzing the microbial composition of 18,340 participants from 24 independent cohorts, the majority of whom were of European ancestry (n = 13,266) (22). A total of 211 taxa (9 phyla, 16 classes, 20 orders, 35 families, and 131 genera) were included in this meta-analysis. The data can be easily accessed from the website (www.mibiogen.org) (23).

### Outcome data sources

GWAS summary statistics for spinal stenosis were obtained from the FinnGen Consortium R8 release (https://r8.risteys.finngen.fi/), which involved 9,169 spinal stenosis cases and 164,682 controls.

### Instrument variables selection

**Figure 2:**
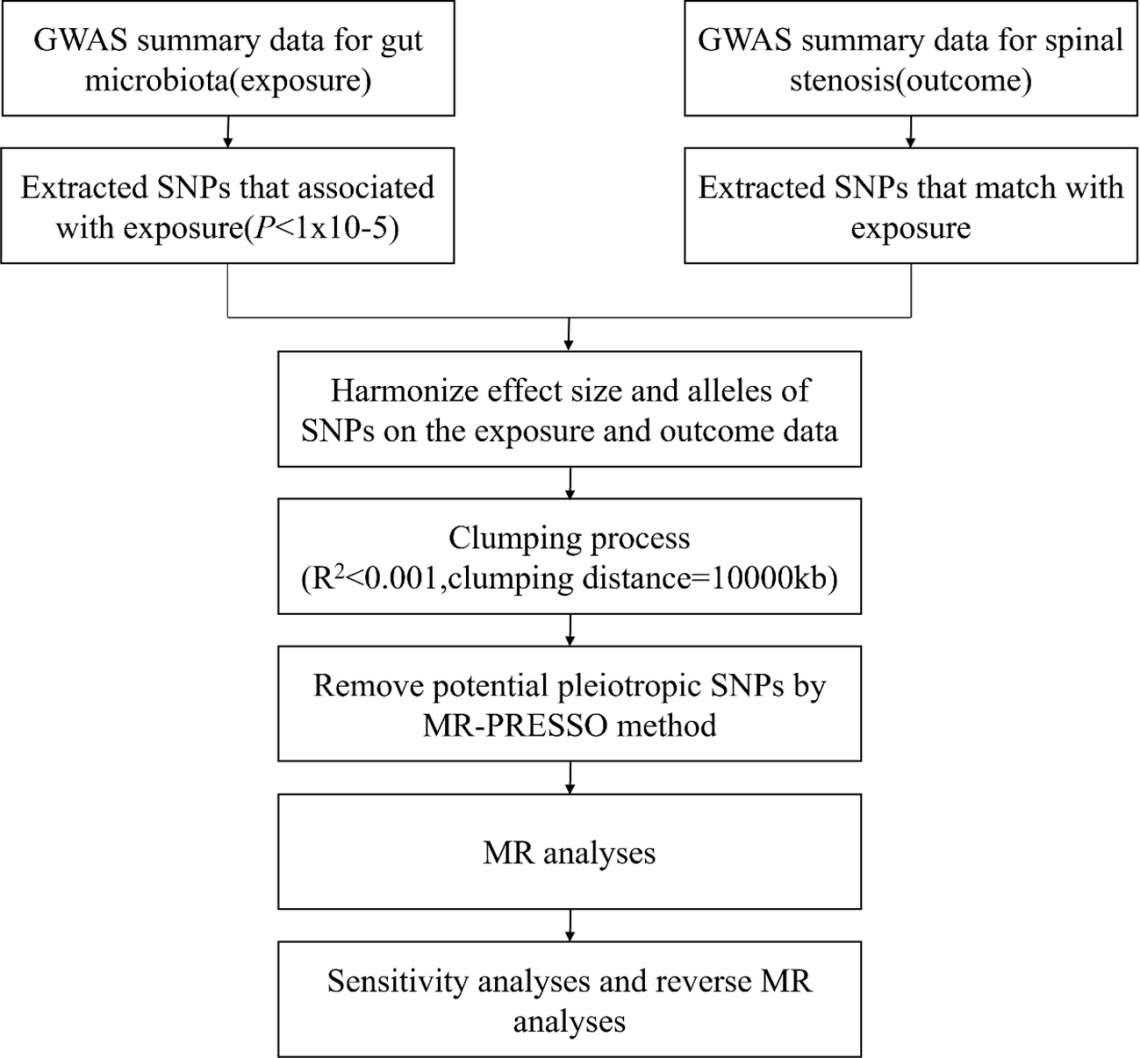
The flow diagram of instrument variables selection.

To ensure the validity and accuracy of the findings on the causal relationship between gut microbiota and spinal stenosis, we employed a rigorous quality control procedure to screen out acceptable IVs. The flow diagram is shown in **Figure 2**.

First, referring to previous MR studies and to ensure that a sufficient number of IVs were acquired, we established the significance level at P < 1.0×10^-5^ to ensure the robustness of the results (24, 25). Second, selected SNPs (R^2^ < 0.001 and clumping distance = 10,000 kb) were clumped to lessen the offset brought on by linkage disequilibrium to guarantee that the IVs are mutually independent (26). Third, we eliminated palindromic SNPs and SNPs that did not appear in the outcome from the IVs (27). Fourth, the PhenoScanner (http://www.phenoscanner.medschl.cam.ac.uk/phenoscanner) database was employed to detect and exclude IVs associated with confounders (educational attainment, smoking behavior, BMI, length of mobile phone use, and watching TV) to meet the principle of independence assumption in MR analysis (28). Finally, we calculated the F statistic to assess the strength of IVs and removed IVs with F < 10 to satisfy the strong correlation with exposure and attenuate instrumental bias (26, 29). F = R^2^(n-k-1)/k(1-R^2^) (n, sample size; k, number of IVs; and R^2^ variance of the exposure explained by the selected SNPs).

### Statistical analysis

The IVW approach (30) was mostly utilized in this MR analysis to provide unbiased estimates of the causal connection between gut microbiota and spinal stenosis. In addition, to estimate the causal relationship under various circumstances, additional techniques were also employed, including the weighted median approach (31), MR-Egger method (32), simple mode method (33), and weighted mode method (34).

To detect potential horizontal pleiotropy effects, we performed the MR-PRESSO and MR-Egger regression intercept analysis. The intercept term in MR-Egger regression can be utilized for assessing horizontal pleiotropy, which if significant demonstrates the presence of horizontal pleiotropy and vice versa indicates the absence of horizontal pleiotropy (35). By eliminating major outliers, MR-PRESSO analysis identifies and decreases horizontal pleiotropy. However, it is important to note that the MR-PRESSO outlier test requires that at least 50% of the genetic variance be valid instrumental variables and relies on the Instrument Strength Independent of Direct Effect (InSIDE) condition (36). After removing pleiotropy, the remaining SNPs were used for subsequent MR analyses.

Cochran’s Q statistic was used to examine the heterogeneity of the SNPs, with a P-value < 0.05 indicating significant heterogeneity (37). Furthermore, to detect possible heterogeneity, we individually removed each instrumental variable SNP and conducted a “leave-one-out” sensitivity analysis to discover if a single SNP conclusively contributed to the causal signal (38). False discovery rate (FDR) corrections were applied to evaluate the false alarm rate. When P < 0.05 but *q*-value ≥ 0.1, gut microbiota and spinal stenosis were considered to have a suggestive association.

Finally, we also performed reverse MR analyses to determine whether reverse causality existed. The “TwoSampleMR” package and the “MRPRESSO” package in the R software (version 4.3.1, https://www.r-project.org/) were used for all MR analyses (36, 39).

## Results

### Instrument variables for gut microbiota

According to a series of filtering steps, a total of 102, 180, 216, 380, and 1363 SNPs at the phyla, class, order, family, and genus levels were identified after a series of filtering steps. After clumping and harmonization, the number of SNPs related to spinal stenosis varies from 3 to 20. Furthermore, the genus *RuminococcaceaeUCG002* contains the highest number of SNPs (20), whereas the genus with the least number is *LachnospiraceaeND3007* (3 SNPs). And no feature, regardless of level, contains a single SNP. **Supplementary Table 1** contains comprehensive details regarding the selected SNPs.

### Causal effects

**Figure 3:**
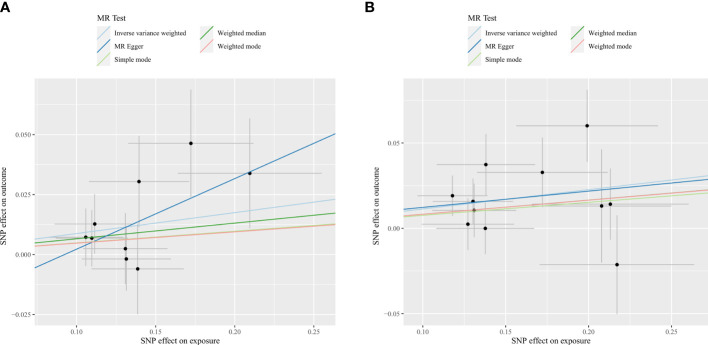
Scatter plots for the causal association between gut microbiota and spinal stenosis. **(A)** genus *Eubacterium fissicatena* group; **(B)** genus *Oxalobacter*.

Initially, our research examined the cause and effect relationship between the gut microbiota and spinal stenosis. Employing the IVW approach as the primary MR detection method, As shown in **Table 1**, **Supplementary Table 2**, and **Figure 3**, the genus *Eubacterium fissicatena* group (OR = 1.09, 95% CI = 1.01-1.18, P = 0.02, IVW) and the genus *Oxalobacter* (OR = 1.12, 95% CI = 1.05-1.20, P = 0.001, IVW) were identified to have a potential causal relationship with spinal stenosis, indicating these two bacterial categories might potentially cause spinal stenosis. Specifically, considering that the above OR values are all greater than 1, the higher genetically predicted *Eubacterium fissicatena and Oxalobacter* levels were associated with a higher risk of spinal stenosis. When the FDR adjustment was carried out, however, these correlations failed to be significant (q > 0.1). Specific IVs can be found in **Table 2**.

**Table 1:** MR estimates for the association between the two gut microbiota and spinal stenosis.

Bacterial taxa (exposure)	MR method	No. of SNP	OR	95% CI	P-value	q-value
*Eubacterium fissicatena* group	MR Egger	9	1.34	0.91-1.99	0.19	0.96
	Weighted median	9	1.07	0.79-1.54	0.20	0.94
	Inverse variance weighted	9	1.09	1.01-1.18	0.02	0.97
	Simple mode	9	1.05	0.90-1.22	0.54	0.96
	Weighted mode	9	1.05	0.90-1.22	0.56	0.99
*Oxalobacter*	MR Egger	11	1.10	0.79-1.54	0.59	0.98
	Weighted median	11	1.09	0.99-1.20	0.09	0.94
	Inverse variance weighted	11	1.12	1.05-1.20	0.001	0.13
	Simple mode	11	1.08	0.92-1.26	0.37	0.96
	Weighted mode	11	1.09	0.95-1.25	0.27	0.99

**Table 2:** SNPs used in MR analysis of the association between the two gut microbiota and spinal stenosis.

Bacterial taxa (exposure)	SNP	Effect allele	Other allele	Exposure (Bacteria)			Outcome (spinal stenosis)			F-statistic
				Beta	SE	P-value	Beta	SE	P-value	
*Eubacterium fissicatena* group	rs10147907	T	G	0.17	0.04	1.36E-05	0.05	0.02	0.04	18.92
	rs11818408	G	A	0.11	0.02	8.04E-06	0.01	0.01	0.55	19.93
	rs11876297	T	C	0.13	0.03	3.06E-06	0.00	0.01	0.89	21.78
	rs151257695	A	G	0.21	0.05	4.10E-06	0.03	0.02	0.14	21.21
	rs1768152	T	C	0.14	0.03	1.03E-05	0.03	0.02	0.11	19.46
	rs2733072	G	A	0.11	0.02	1.57E-06	0.01	0.01	0.56	23.06
	rs3771393	C	T	0.13	0.03	9.27E-07	0.00	0.01	0.87	24.07
	rs6934739	A	G	0.11	0.03	1.04E-05	0.01	0.01	0.31	19.44
	rs7104872	G	A	0.14	0.03	2.05E-06	-0.01	0.02	0.75	22.54
*Oxalobacter*	rs11108500	A	G	-0.20	0.04	3.17E-06	-0.06	0.02	0.00	21.70
	rs111966731	T	C	0.21	0.05	6.22E-06	0.01	0.02	0.50	20.42
	rs12002250	A	C	0.22	0.05	3.22E-06	-0.02	0.03	0.46	21.68
	rs1569853	T	C	-0.14	0.03	3.33E-06	-0.04	0.02	0.04	21.61
	rs36057338	G	T	0.21	0.04	8.15E-07	0.01	0.03	0.69	24.32
	rs3862635	C	T	-0.17	0.04	1.25E-05	-0.03	0.02	0.11	19.08
	rs4428215	G	A	0.13	0.02	7.50E-08	0.02	0.01	0.24	28.93
	rs6000536	C	T	-0.13	0.03	2.45E-07	-0.01	0.02	0.50	26.63
	rs6993398	G	A	0.13	0.03	5.06E-06	0.00	0.02	0.87	20.81
	rs736744	C	T	0.12	0.02	2.41E-08	0.02	0.01	0.11	31.13
	rs115602804	G	A	0.10	0.02	3.69E-06	0.00	0.02	0.91	21.41

Among the two causal correlations revealed above, the F-statistics of the IVs were larger than 10, eliminating the bias of weak IVs (**Table 2**; **Supplementary Table 1**). Cochran’s IVW Q-test indicated that no significant heterogeneity was observed in the IVs of the genus *Eubacterium fissicatena* group (P = 0.67) and the genus *Oxalobacter* (P = 0.45) (**Supplementary Table 3**). In addition, according to the results of the MR-Egger regression intercept analysis, no significant directional horizontal pleiotropy was identified either in the IVs of the genus *Eubacterium fissicatena* group (P = 0.33) and the genus *Oxalobacter* (P = 0.92) (**Supplementary Table 4**). In addition, none of the above SNPs revealed a potential relationship with confounders (educational attainment, smoking behavior, BMI, length of mobile phone use, and watching TV) associated with spinal stenosis (28).

According to the results of reverse MR analysis, no significant causal association was found between spinal stenosis and the mentioned gut microbiota, the genus *Eubacterium fissicatena* group (OR = 0.94, 95% CI = 0.87-1.03, P = 0.19, IVW) and the genus *Oxalobacter* (OR = 0.96, 95% CI = 0.89-1.04, P = 0.31, IVW) (**Table 3**). Promising results (P > 0.05) were obtained for heterogeneity based on the Cochran’s IVW Q-test and directional horizontal pleiotropy based on the MR-Egger regression intercept analysis. In addition, relevant information on the selected IVs and MR results about reverse MR analysis can be found in **Supplementary Table 5**.

**Table 3:** Reverse MR analysis between spinal stenosis and the two gut microbiota.

Bacterial taxa (outcome)	MR method	No. of SNP	OR	95% CI	P-value	q-value
*Eubacterium fissicatena* group	MR Egger	80	1.06	0.68-1.64	0.80	0.95
	Weighted median	80	0.96	0.85-1.09	0.50	0.60
	Inverse variance weighted	80	0.94	0.87-1.03	0.19	0.31
	Simple mode	80	1.00	0.73-1.36	0.98	0.98
	Weighted mode	80	0.98	0.75-1.28	0.87	0.87
*Oxalobacter*	MR Egger	49	0.99	0.67-1.45	0.95	0.95
	Weighted median	49	0.97	0.87-1.08	0.60	0.60
	Inverse variance weighted	49	0.96	0.89-1.04	0.31	0.31
	Simple mode	49	1.10	0.81-1.48	0.55	0.98
	Weighted mode	49	1.14	0.86-1.51	0.35	0.87

Moreover, in at least one MR method excluding IVW, eight bacterial taxa, the class Clostridia (OR

**Figure 4:**
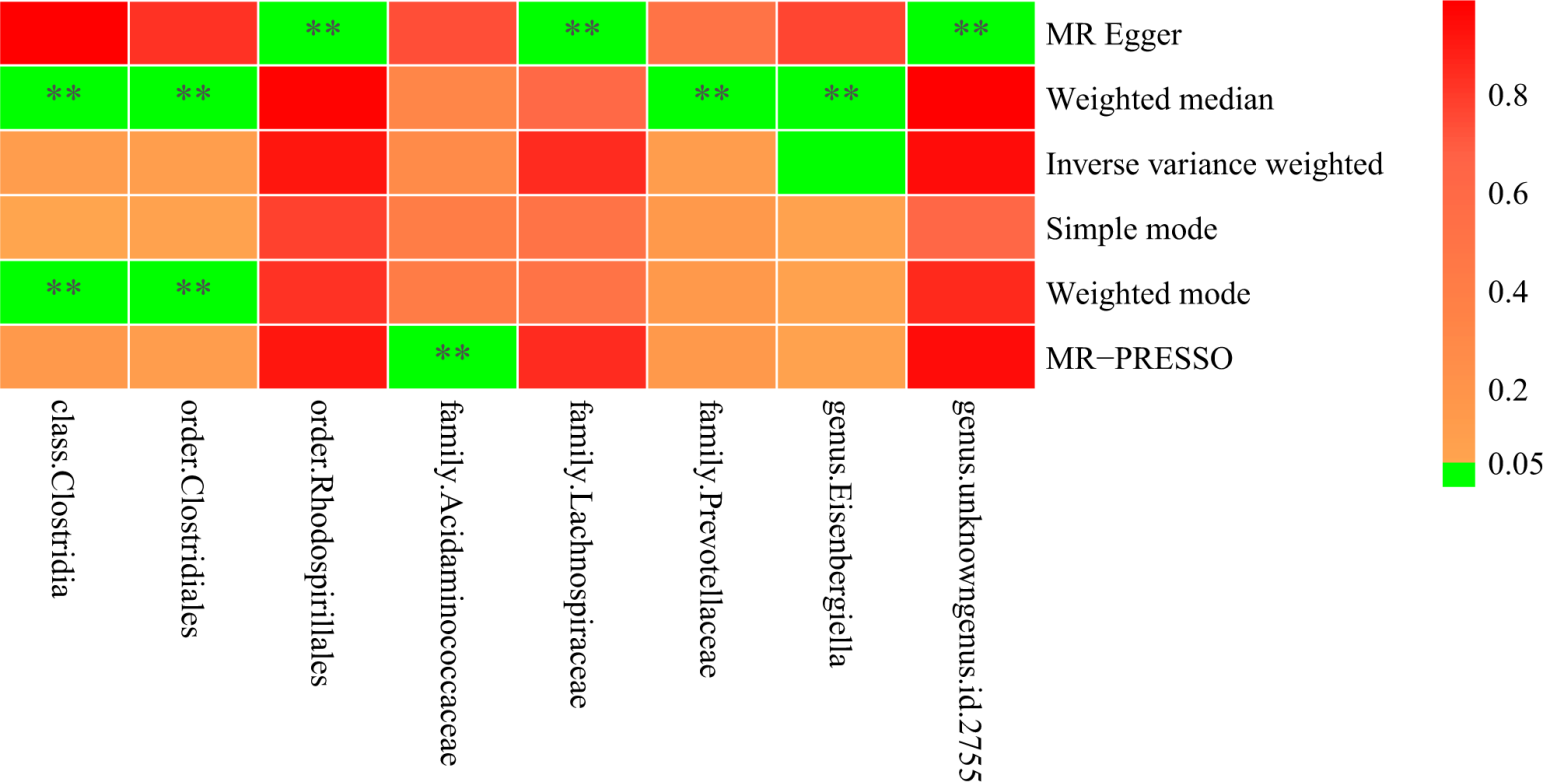
Heatmap illustrating eight bacterial taxa were revealed to be suggestively related with spinal stenosis, with ** indicating P < 0.05.

= 1.39, 95% CI = 1.14-1.70, p = 0.001, Weighted median; OR = 1.43, 95% CI = 1.09-1.87, P = 0.03,

Weighted mode), the order Clostridiales (OR = 1.37, 95% CI = 1.13-1.65, P = 0.001, Weighted median;

OR = 1.40, 95% CI = 1.08-1.81, P = 0.02, Weighted mode), the order Rhodospirillales (OR = 1.46, 95% CI = 1.05-2.03, P = 0.04, MR Egger), the family Lachnospiraceae (OR = 1.65, 95% CI = 1.06-2.51, P = 0.03, MR Egger), the family Prevotellaceae (OR = 0.86, 95% CI = 0.75-0.98, P = 0.03, Weighted median),

the family Acidaminococcaceae (Causal Estimate = -0.07, SD = 0.03, T = -2.65, P = 0.04, MR-PRESSO), the genus Eisenbergiella (OR = 0.87, 95% CI = 0.78-0.97, P = 0.03, MR Egger), the genus unknowngenus.id.2755 (OR = 1.49, 95% CI = 1.10-2.02, P = 0.03, MR Egger), were revealed to be suggestively related with spinal stenosis (**Figure 4**; **Supplementary Tables 2**, **6**).

### Sensitivity analyses

**Figure 5:**
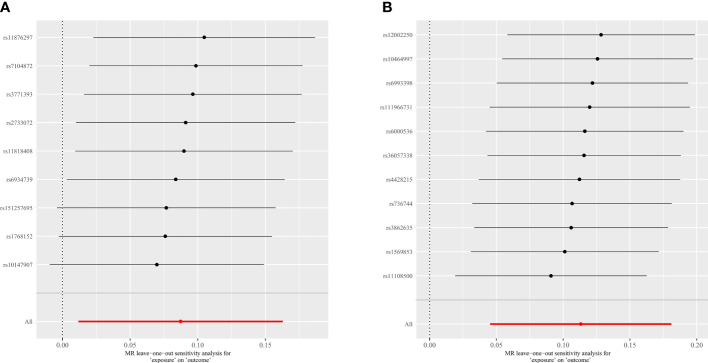
Leave-one-out plots for the causal association between gut microbiota and spinal stenosis. **(A)** genus *Eubacterium fissicatena* group; **(B)** genus *Oxalobacter*.

In this study, Cochran ‘s IVW Q-test was utilized to evaluate heterogeneity, and the MR-Egger regression intercept analysis was used to evaluate directional horizontal pleiotropy. Furthermore, we employed MR PRESSO to ensure global directional horizontal pleiotropy and increase the reliability of the study (**Supplementary Table 6**). At the level of five different strains, MR PRESSO was performed for each intestinal flora except the genus *LachnospiraceaeND3007* because there were insufficient SNPs (n ≤ 3) to conduct MR PRESSO. And one of the class levels, one of the order levels, three of the family levels, and eighteen of the genus levels were tested for outlier-corrected MR-PRESSO to ensure the robustness of the MR results (**Supplementary Table 6**). In addition, the leave-one-out analyses were performed to examine the potential influence of individual SNPs on the observed associations. As shown in **Figure 5**, when an SNP is eliminated individually, the overall error lines are all positioned on one side of the median line, implying that each SNP affects the results equally and excludes outlier SNPs from affecting the reliability of the results.

## Discussion

Recently, researchers have revealed that the composition and variety of gut microbial components are closely linked to spine-related disorders such as osteoporosis (11), intervertebral disc degeneration (40), spinal sarcopenia (41), adolescent idiopathic scoliosis (42), ankylosing spondylitis (43), and others. An article, based on previous research, innovatively proposes the concept of the gut-spine axis by reviewing articles on gut microbiota and spine-related diseases (13). In terms of the association between gut microbiota and spinal stenosis, Tadatsugu Morimoto et al. demonstrated that a Gut-ligament axis may be present in lumbar spinal stenosis, but the specific connection is unknown (13). In this study, a two-sample MR analysis was performed to assess the causal relationship between gut microbiota and spinal stenosis, employing summary statistics for gut microbiota from the largest GWAS meta-analysis carried out by the MiBioGen consortium and summary statistics for spinal stenosis from the FinnGen consortium R8 release data. With the help of large-scale GWAS data sources, our MR study fills this knowledge gap from a new perspective. To the best of our knowledge, this is the first MR study to investigate the potential causal relationship between gut microbiota and spinal stenosis. Interestingly, the finding indicates that genetically predicted abundance of specific gut microbiota (the genus *Eubacterium fissicatena* group and genus *Oxalobacter*) is potentially associated with spinal stenosis. Additionally, we also identified some gut microbiota may be potentially related to spinal stenosis. Importantly, the above results may have implications for public health efforts to minimize the risk of development of spinal stenosis.

*Eubacterium fissicatena*, growing at 20–40 °C (optimum 37 °C) and pH 6.0–8.0 (optimum pH 7.0), was isolated from the alimentary tract of the goat as first reported by M. Taylor in 1972 (44). Eubacterium, regarded as promising targets for microbial therapeutics, forms the core genera of health-associated human gut microbiota (45). Over the past few years, an increasing number of studies have shown that *Eubacterium* modulates immune and inflammatory responses in humans through the regulation of short-chain fatty acids (SCFAs) which have significant effects on gut health (45, 46). In addition to its anti-inflammatory effects, butyrate, the primary metabolite of SCFAs in the colon, is necessary for the maintenance of intercellular tight junctions, which preserves the normal barrier function of intestinal mucosa (47). Several studies conducted in mice have shown that specific medication components or metabolites can modulate dextran sulfate sodium salt-induced colitis by correcting intestinal flora dysbiosis (decreasing the abundance of pathogenic bacteria, *Eubacterium fissicatena*) (48–50). Furthermore, in terms of metabolism-related diseases, the reduction of *Eubacterium fissicatena* abundance is effective in reducing diet-induced obesity, hepatic steatosis, dyslipidaemia, and insulin resistance (51–54). Taken together, the existing research indicates that *Eubacterium fissicatena* is most often a causative agent in the mediation of inflammation-related diseases and metabolic disorders, including obesity and dyslipidaemia. As mentioned in the background section, there is a correlation between these factors and spinal stenosis, and what seems to serve as an explanation for the existence of a possible causal relationship between *Eubacterium fissicatena* and spinal stenosis.

In contrast to *Eubacterium fissicatena*, *Oxalobacter*, first reported as a novel anaerobic bacterium for degrading oxalic acids in 1985, is a popular species of interest to researchers in our literature search (55). Since the separation of *Oxalobacter*, numerous studies have concentrated on its role in hyperoxaluria and kidney stone development and attempted to develop associated therapies to limit the formation and advancement of the above-mentioned diseases, owing to its specific function in degrading oxalic acid (56–59). OxlT, an oxalate transporter protein of *Oxalobacter*, specifically absorbs oxalate from the intestine into bacterial cells, lowering the likelihood that the host animal may develop oxalate-deposition illnesses such as kidney stones (60). Remarkably, when examining the connection between *Oxalobacter* and spinal stenosis, we discovered a case report by Knight RQ et al. suggesting that lumbar spinal stenosis could potentially be caused by oxalic acid accumulation (61). As a supplement, the correlation between *Oxalobacter* and spinal stenosis at the genetic level was explored in the present MR study to enhance insights in this field.

Furthermore, we found eight strains of organisms that might be related to spinal stenosis based on five MR statistical approaches other than the IVW method, in addition to the two strains (the genus *Eubacterium fissicatena group* and the genus *Oxalobacter*) that were obviously causally linked to the condition. As mentioned above, eight bacterial taxa, 1 class (*Clostridia*), 2 orders (*Clostridiales, Rhodospirillales*), 3 families (*Lachnospiraceae, Prevotellaceae, Acidaminococcaceae*), and 2 genera (*Eisenbergiella, unknowngenus.id.2755*), may have a potential relationship with spinal stenosis. However, to confirm the aforementioned association between gut microbiota and spinal stenosis, large-scale population sequencing studies need to be performed.

Overall, this study has several strengths. Firstly, employing MR analysis, we established the genetic causal relationship between gut microbiota and spinal stenosis, and importantly identified two gut microbiota (the genus *Eubacterium fissicatena* group and genus *Oxalobacter*) that were causally linked to the condition. This work contributes to the body of knowledge in this field and holds the potential to contribute to public prevention strategies for spinal stenosis. Second, to improve the robustness of the study findings, MR-PRESSO and MR-Egger regression intercept terms were utilized to test for horizontal pleiotropy. Furthermore, to avoid bias, the two-sample MR study adopted non-overlapping exposure and outcome summary data (62).

Nevertheless, there are several limitations regarding our study that should be acknowledged. First, as the majority of participants of the aggregated data in this research were of European ancestry, the extent to which findings from this study can be extrapolated to other ethnic groups may be limited. Second, following the relevant research papers, we set the significance level for SNPs to P < 1 × 10^-5^ to acquire more instrumental variables, which implies that SNPs employed in the analyses in the current study did not satisfy the standard GWAS significance threshold (P < 5 × 10^-8^) (19, 22). For this, we carefully evaluated the F-value of each SNP to eliminate the potential effect of weak IVs bias. Furthermore, the majority of individuals with spinal stenosis are middle-aged or older, while our current study is unable to divide the intestinal flora based on aging.

## Conclusions

In summary, we comprehensively assessed the causal association between the gut microbiota and spinal stenosis. As a result, the genus *Eubacterium fissicatena* group and genus *Oxalobacter* are considered to have a potentially genetic causal relationship with spinal stenosis. Undoubtedly, this study might provide new insights into the pathogenesis of gut microbiota-mediated spinal stenosis. However, further large-scale clinical trials are needed to confirm these findings and to develop public prevention strategies for spinal stenosis, as well as to explore the potential of targeted probiotics for the treatment of spinal stenosis.

## Data availability statement

The original contributions presented in the study are included in the article/**Supplementary material**. Further inquiries can be directed to the corresponding authors.

## Author contributions

JL: Conceptualization, Data curation, Investigation, Methodology, Software, Writing – original draft. JPW: Writing – original draft, Data curation, Formal analysis, Visualization, Software. JNW: Conceptualization, Investigation, Software, Writing – original draft. TX: Conceptualization, Data curation, Writing – original draft. BW: Investigation, Visualization, Writing – original draft. SY: Investigation, Software, Writing – original draft. SJ: Methodology, Writing – original draft. HW: Writing – review & editing. HH: Writing – review & ,editing Supervision.

## Supplementary material

Supplementary Table 1 Instrumental variables used in MR analysis of the association between gut microbiota and spinal stenosis.

Supplementary Table 2 Full result of MR estimates for the association between gut microbiota and spinal stenosis.

Supplementary Table 3 The heterogeneity of gut microbiota instrumental variables.

Supplementary Table 4 Directional horizontal pleiotropy assessed by intercept term in MR Egger regression of the association between gut microbiota and spinal stenosis.

Supplementary Table 5 The results of reverse MR analysis between spinal stenosis and gut microbiota.

Supplementary Table 6 MR-PRESSO analysis for the association between gut microbiota and spinal stenosis.
